# Israel’s 2008 Organ Transplant Law: continued ethical challenges to the priority points model

**DOI:** 10.1186/s13584-018-0203-6

**Published:** 2018-03-16

**Authors:** Corinne Berzon

**Affiliations:** Bar Ilan University, Ramat Gan, Israel

**Keywords:** Organ procurement, Organ allocation, Priority, Israel, Routine retrieval

## Abstract

In 2008, responding to a widening gap between need and availability of transplant organs, Israel’s Ministry of Health adopted a program of incentivized cadaveric organ donation. The Organ Transplant Law rewards individuals with prioritized access to organs on the condition that they participate in procurement efforts. Priority is awarded in the form of additional points allocated to the individual’s organ recipient profile. Although Israel has experienced moderate gains in the years since the law’s implementation, these have not been sufficient to satisfy the demand. Furthermore, the law faces logistical and ethical challenges. These challenges could potentially be resolved by shifting the organ procurement default to routine retrieval rather than the current default of presumed refusal to organ retrieval.

This paper examines philosophical and practical challenges to the priority points policy and weighs whether Israel should consider an alternative policy of routine retrieval of transplant organs with the option to opt out of the donor pool.

## Introduction

It is estimated that less than 1.5% of people who die meet the eligibility criteria for organ donation. Since the implementation of brain stem testing in the 1970’s, the principal source of cadaveric transplant organs has been brain dead donors who have voluntarily signed donor cards, the consequence of which is that the number of patients awaiting organ transplants is increasing while the supply of viable organs meets only a fraction of the demand. While there is an organ gap throughout the world, it is especially marked in Israel where religious and cultural barriers exacerbate the problem. The inadequate supply of organs in Israel in the past led needy patients to turn to transplant tourism, traveling abroad to purchase kidneys on the black market. In 2008, a group of physicians, ethicists, and politicians put forth an innovative new law that would halt Israeli access to foreign organs while providing incentives to donate both live and cadaveric organs. Although transplant tourism has been effectively eradicated and live donation has increased dramatically, in part because of the support of private organizations, increasing the availability of cadaveric transplant organs remains a challenge. Furthermore, ethical reservations regarding the law remain unresolved nearly a decade since its adoption. This paper describes these reservations and possible responses to them, and briefly explores potential alternatives to the 2008 Organ Transplant Law.

## Background: Israel’s 2008 Organ Transplant Law

The Organ Transplant and Brain-Respiratory Death Law responded to three major challenges to organ procurement in Israel: 1) confusion regarding determination of death, 2) organ trafficking and unethical transplant tourism, and 3) the critical dearth of transplantable organs – both live and cadaveric [1].

### Brain-respiratory death

Responding to misconceptions and inconsistencies regarding brain death determination, the first section of the law attempted to provide a legal definition of time of death that would satisfy both medical and religious requirements. However, the 2008 Brain-Respiratory Death Law has met with challenges. One challenge is that physicians with the authority to determine death are required to undergo a voluntary training course. Since the law’s implementation, fewer than half of the number of physicians are certified in the new standards of brain death determination than were prior – dropping from 210 to 102 [2, 3]. This decrease resulted in fewer brain death determinations in the years following the implementation of the law, or in delays that compromised organ retrieval for the purposes of transplantation. Another logistical obstacle relates to novel standards of ancillary and apnea testing. After the law was passed, some hospitals were unable to determine brain death in accordance with the law because they did not possess the correct apparatus required for the stipulated tests [2, 3]. Though the necessary equipment is now available in most hospitals, the demanding testing process and lack of certified doctors contributes to delays and limit the number of brain death determinations. This, in turn, affects rates of organ retrieval.

Another obstacle posed by the Brain-Respiratory Death Law is that it requires physicians to consult with families of potential donors prior to determining brain death. Since many families resist brain death testing, fewer brain death determinations are ascertained, resulting in lower availability of transplant organs [2, 3]. While this paper will not focus on brain death determination, it is important to note that this aspect of the law has been detrimental to efforts at increasing organ donation.

The second part of Israel’s 2008 Organ Transplant Law consists of three initiatives designed to increase both live and cadaveric donation on the one hand, and eliminate unethical transplant tourism and organ trafficking on the other.

### Transplant tourism

**Fig. 1:**
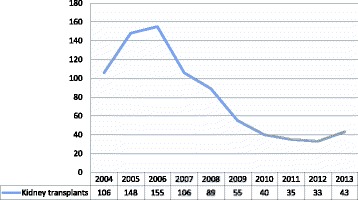
Kidney transplants obtained abroad by Israeli recipients. (source: Ashkenazi, T., Lavee, J. & Mor, E. Organ donation in Israel – achievements and challenges. *Transplantation* 2015;99(2):265–266)

This section of the law bans organ trafficking and reimbursement by Israeli health funds for illegal transplant tourism. For years, in the face of the severe organ shortage, Israeli authorities tolerated the practice of purchasing organs abroad and facilitated transplant tourism by permitting local health funds to reimburse patients for commercially transplanted organs [4–6]. The 2008 Organ Transplant Law stipulates that transplants performed outside of Israel must be conducted in accordance to the laws of the host country and must also comply with Israeli law. Insurance companies are only permitted to reimburse if transplants are performed within the boundaries of these regulations. This section of the law has succeeded in curbing transplant tourism [Fig. 1].

### Live donation incentives

The second section of the Organ Transplant Law removes disincentives for live donation. Through multiple forms of reimbursement, live altruistic or directed donors may donate a kidney, bone marrow, or a liver splice without suffering undue financial strain. While this may have contributed to increasing rates of live organ donation in Israel, live donation has also become a popular form of charity among Orthodox Jews. The organization *Matnat Chayim* (‘Gift of Life’) has facilitated 463 kidney donations since 2009 with nearly all donors identifying as Orthodox or ultra-Orthodox [7]. The total number of live altruistic kidney donors in Israel from 2009 to 2016 was 1032, meaning that approximately 40% of live kidney donations in Israel since 2009 have been facilitated by *Matnat Chayim* [7]. Thus, while the Organ Transplant Act has removed disincentives from live donation and has constrained the ability for Israelis to seek kidney transplants abroad, charitable kidney matching has greatly contributed to the increase in live organ donation over the same time period that the law has been in effect.

### Priority points

The third section of the Organ Transplant Law offers three degrees of priority in the form of additional points allocated to a patient’s recipient profile should they require an organ transplant. The priority points policy became operative in April of 2012.

1. *Maximum* priority is extended to patients who have a first-degree family member who was a cadaveric donor, or have themselves previously been live donors [8]. Maximum priority takes immediate effect with no waiting period.
2. *Regular* priority rewards registered donors of at least 3 years, although this waiting period was initially waived to encourage greater public participation when it was first introduced in 2011. Priority points act as a tie breaker by giving an advantage to consented donors over non-donors in cases of similar medical need [9].
3. *Second* priority awards a small number of points to individuals with first-degree relatives who are registered as an organ donor [10].

**Fig. 2:**
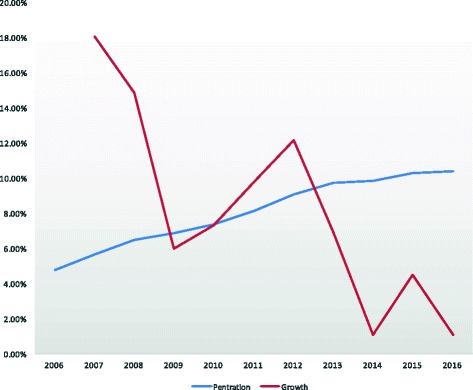
Growth/penetration of ADI card. (source: www.adi.gov.il/קצת-עלינו/סיכום-פעילות/סיכום-פעילות-המרכז-הלאומי-להשתלות-2016/)

Israel’s priority policy is unprecedented and innovative. While a priority system is in place in Singapore, it was implemented alongside routine retrieval – individuals who choose to exit the organ pool are deprioritized on the organ transplant waiting list [11]. In this sense, increases in authorization cannot be separated from the routine retrieval policy. The Israeli legislation, contrarily, preserved the pre-existing voluntary procurement policy and applies priority to those who join the pool of organ donors. Widespread media campaigns accompanying the implementation of the Israeli Organ Transplant Law have increased public awareness of the organ gap and encouraged more Israelis to sign donor cards [12]. The increase in new card carriers was especially marked from 2011 to 2013 during the implementation period, but has since dropped off [Fig. 2]. This indicates that media campaigns may have been instrumental in encouraging consent to donation rather than the incentive program itself. Currently nearly 900,000 Israelis, amounting to approximately 14% of the adult population, have signed an organ donor card and are eligible for priority [13].

**Fig. 3:**
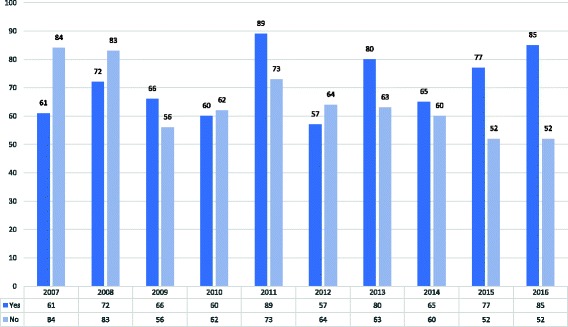
Consent to actual retrieval by next of kin. (source: www.adi.gov.il/קצת-עלינו/סיכום-פעילות/סיכום-פעילות-המרכז-הלאומי-להשתלות-2016/)

**Fig. 4:**
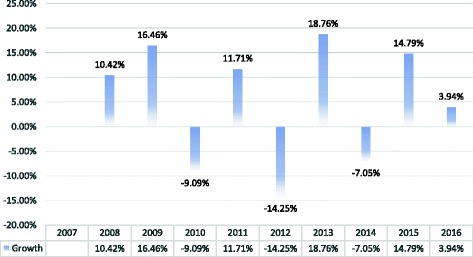
Year over year growth in consent to retrieval. (source: www.adi.gov.il/קצת-עלינו/סיכום-פעילות/סיכום-פעילות-המרכז-הלאומי-להשתלות-2016/)

Studies have shown that when next of kin know that the decedent consented to organ donation they will likely not block retrieval [14, 15]. By encouraging Israelis to sign a donor card during their lifetime, more families are aware of the decedent’s wishes and are more likely to authorize retrieval. In 2016 the rate of family consent to organ retrieval reached 62%, a 20% from the 2007 consent rate prior to the Organ Transplant Law [Figs. 3 and 4]. While these gains are significant, Israel continues to lag behind Europe and North America in consent to cadaveric organ retrieval. In 2016, the donation rate per million of population (PMP) was just 10 deceased donors PMP [16] and donor registration was far lower than in Europe and North America. Therefore, while Israelis are benefitting from greater awareness and authorization to organ donation, maximizing organ retrieval continues to pose a challenge. Furthermore, ethical challenges to the priority model have been raised. Thus, while the Organ Transplant Law succeeded in increasing consent to donation by next of kin and raised awareness of organ donation in general, further examination is warranted.

## Review of challenges to Israel’s priority policy

Since the law’s implementation, positive effects of the priority system have been recorded. However, the policy has also been met with criticism. Several articles have been published challenging priority allocation in general and the Israeli formulation in particular. In the following section, I will summarize challenges to Israel’s priority policy and possible responses to them.

### Non-medical criteria should not determine the allocation of scarce medical resources

It has long been accepted that organ transplantation resource allocation should be performed primarily in accordance to medical criteria [17, 18]. Prioritizing allocation of transplant organs to individuals based on participation in the organ pool directly contravenes this policy by introducing an ethical criterion. Yechiel Bar Ilan highlights this difficulty in a 2014 article in *Harefua*. Bar Ilan asks why, if it is justifiable to allocate organs based on participation, would other limited medical resources not likewise be distributed per ethical criteria [19]? For example, beds in critical care units could be allocated first to non-smokers or individuals who volunteered their time or money to Israel’s medical system. Blood donation could likewise be distributed with priority to those who have donated in the past. Furthermore, if moral reasons are considered as a factor in organ allocation, why should these be limited to signing an organ donor card? Perhaps previous drug, alcohol or tobacco addiction, obesity, or criminal convictions [20] should likewise factor in the allocation process. The widespread consensus is that moral blame should not disqualify - or deprioritize - individuals from receiving medical care, and the Israeli policy contravenes this consensus.

One possible response to this challenge is that since priority only functions as a tie-breaker between cases of similar clinical need, preferred status does not explicitly contravene the distribution of scarce medical resources in accordance to medical need [21]. However, since the implementation of Israel’s priority policy dozens of potential recipients have been bypassed by card carriers. If medically relevant factors were the only ones applied, the organ may have been allocated first to the non-card carrier. In this sense, the determining factor is consent to donation. In such cases, one person’s prioritization is another person’s deprioritization and as such is punitive. Non-card carriers are punished for their lack of participation, whether this lack of participation is a conscious decision or a lack of initiative. Bar-Ilan describes this as an infringement on freedom of expression and conscience [19]. If signing an organ card is voluntary, then it should not be attached to punishment and reward - receiving medical care based on need is a basic right and therefore should not be contingent on participation or contribution.

A response to this argument is that the distribution of scarce medical resources should not be performed solely in accordance to medical criteria. Although organs are a scarce medical resource, they are not a typical good - their provision rests on the willingness of individuals to give their body parts to others. In this sense, the success of the entire transplantation enterprise depends on collective participation. When individuals are willing to receive organs but are not willing to give them, this lack of reciprocity justifies prioritizing ‘givers’ over ‘takers’. Robertson, in his paper on priority allocation, calls this a “cooperative system” and states that allocating organ first to individuals who instantiate the system is a matter of justice [22]. Permitting non-donors, or “free riders”, equal access violates the principle of reciprocity and as such is unjust. However, like the Singapore model, Robertson’s proposal refers to deprioritizing non-donors in a routine retrieval system [22]. By limiting what Bar Ilan refers to as the punitive aspects of priority allocation to those who have actively chosen to opt-out, routine retrieval with deprioritization avoids punishing individuals who may not have joined the organ pool out of ignorance or lack of opportunity. By including all individuals in the pool of prioritized potential organ donors, the rate of donation may be sufficient to offset the punitive aspects of priority altogether: if enough people remain in the donor pool, even those who choose to opt-out with diminished priority will have greater access than in a voluntary procurement system. Stephanie Eaton writes: “The practical consequences of opting-out would entail that the person who is a free-rider is liable to be discriminated against in the allocation of organs” [23]. While these incidences of priority allocation would be infrequent, the policy would contribute to public discourse on solidarity and the importance of remaining in the organ pool. For Eaton, like Robertson, the ideal policy is one of routine retrieval bolstered by disincentives to discourage opting out. The equalizing effect of routine retrieval is absent in the Israeli model; potential organ recipients are passed over even though they have not deliberately exited the donor pool. Bar Ilan describes this as ironic because voluntary organ donation emphasizes altruistic giving and not obligation. By doling out “altruistic punishment” to non-donors, the Israeli law contravenes basic human dignity and freedom [19].

Priority allocation departs from the principle that distribution of scarce medical resources be in accordance to medical criteria [24]. This departure is amplified in an opt-in model, such as Israel’s, where large numbers of people do not register out of apathy and ignorance rather than deliberate avoidance. Priority allocation leaves these inadvertent free-riders at a disadvantage because of passive inaction rather than deliberate refusal. While Robertson and Eaton justify deprioritization in a routine retrieval system where refusers must deliberately exit the pool, the policy is punitive in the context of voluntary procurement. Furthermore, as Bar Ilan states, if altruistic donation is the underlying procurement policy, punishing individuals who do not behave altruistically contravenes freedom of conscience and expression: priority allocation undermines the voluntariness of altruistic donation.

### The policy rewards individuals for the behaviour of their family members

Distribution of scarce medical resources in accordance to moral deservingness is contrary to the principle that scarce medical resources be allocated solely by need. However, incentivization may be viewed as a reward for participation in a reciprocal enterprise: those who promise to contribute are promised greater potential benefit. However, the Israeli law goes further by including the registration of family members as a basis for priority. This aspect of the law cannot be defended as an in-kind exchange and is therefore problematic. As Quigley, M. et al. explain in their comprehensive analysis of Israel’s law: “The pertinent point is not whether individuals should benefit from their own good actions, that is, signing their own donor card, but whether they should benefit from the good actions of others where they themselves have not signed a donor card” [25]. In this sense, it is not the reciprocal behaviour of the individual that is being rewarded but that of their relatives, and therefore appeals to justice are inapplicable.

By assigning priority to individuals who have first degree relatives that have signed donor cards, the law faces the added challenge of unfairly benefitting individuals from large families. As Quigley, et al. point out, those with no first-degree relatives are excluded from this level of priority entirely, and those with fewer siblings have lower chances of gaining it [25]. Considering that individuals with many siblings already have a greater chance of finding a tissue-matched living donor, giving priority to individuals whose family members are consented donors is unnecessarily prejudicial. These individuals could consent to donation themselves and receive a higher degree of priority, as stated by Lavee, et al. in response to this criticism: “[…] Everyone is welcome to sign their own card, thereby ensuring themselves higher priority than those gained by a relative’s signature on the donor card” [26]. However, this “misses the ethical nub of the matter” [25]. The question is whether it is justifiable to reward individuals whose relatives have signed a donor card, not whether they may gain greater reward through their own good acts.

Another response to the criticism of proxy priority is that if the model induces high enough rates of donation then even those disadvantaged by small families will be better off. As Lavee, et al. explain: “If the Israeli law achieves its goal of obtaining more organs for transplantation, everyone, including people with fewer first-degree relatives, will benefit” [26]. However, this level of donation has not been reached, and once again this response “misses the ethical nub of the matter”. Even if individuals disadvantaged by the law stand to be better off, this does not resolve the challenge that family size should not be a factor in the allocation of transplant organs.

The Israeli law offers maximum priority for individuals who have authorized organ retrieval from a first-degree relative. These priority points are apportioned without the three-year waiting period stipulated by regular priority. This instant and significant priority is likewise granted to individuals who have themselves been live donors. Quigley, et al. point out that the cost of live donation is greater than the cost of permitting the retrieval of a relative’s organs, and therefore the allocation of priority points should not be equivalent [25]. Furthermore, this level of priority also favours individuals from large families who have greater opportunity to authorize retrieval of cadaveric organs. Although it is possible for individuals to gain regular priority by signing their own donor card, this degree of priority requires a three-year waiting period and is less advantageous. While advantage should be offered to live donors in proportion to their contribution, offering the same benefit to individuals with first-degree relatives who have been cadaveric donors is problematic both because it offers greater advantage to individuals from larger families and because it rewards individuals for the contribution of others.

### The policy enables pernicious gaming

Another problematic effect of granting maximum priority to next of kin is that families may be encouraged to donate their loved ones’ organs against the decedent’s wishes to gain priority. This challenge is highlighted in Silva and Wright’s critique of the clause [27]. Considering that potential gains are immediate, there is an incentive to game the system. This gaming would result in organs being retrieved from non-consenting decedents. The law is further vulnerable to another loophole. As explored by Kessler and Roth in a series of experimental games, the model permits gaming by not making consent to donation legally binding, thereby allowing individuals to gain priority while instructing next of kin not to authorize donation. Opening this loophole could reduce the number of consented donors and negatively impact transplant sustainability [28]. The card also gives registrants the option to check a box requesting that a cleric be consulted before organ donation occurs. An individual who wants priority but does not wish to donate their organs could check that box with the implicit or explicit understanding that their chosen clergyperson would refuse donation. While there is no evidence that such gaming has occurred in Israel, Kessler and Roth have shown that loopholes may lower trust in the system. One possible resolution to this challenge is to make active, ante mortem consent to donation legally binding so that family or clergy cannot override the wishes of potential donors. While this would potentially solve the problem of the loophole, it is unlikely that organs would ever be retrieved in face of family opposition. Therefore, even if an individual has signed a donor card as an expression of their wishes, in practice this expression is unenforceable.

### The law permits next of kin to defeat the express wishes of signed donors

Israel’s ADI organ donor card stipulates several options: individuals may choose to donate their organs without exception or they may exclude specific organs from retrieval, and may additionally require that family or religious authorities be consulted prior to retrieval. In practice, however, no procedures will be performed if the family protests. By extending the final decision to family members or religious authorities regardless of the decedent’s express wishes, the law fails to respect the wishes of signed donors.

Voluntary organ procurement policies emphasize explicit consent to donation. The retrieval of organs against the ante mortem wishes of the decedent is regarded as wrong or unethical. In the absence of explicit consent, for example if there is no donor card and no family is available to offer proxy consent to retrieval, organs will not be retrieved. The belief that autonomy and consent are “central not only to organ procurement, but to the practice of medicine in general” [29] has led to the prominence of voluntary organ retrieval policies. However, it is unclear how opt in systems that permit families to override the decedent’s express wishes to donate cohere with this understanding of consent [30]. Upon closer examination, it is not the individual’s consent that is respected, but that of their families [31]. The difficulty of permitting family to override the explicit wishes of the decedent in the Israeli model was highlighted in 2011 when family members of a popular Israeli footballer overrode his consent to organ donation [32]. If consent to donation is not binding then the donor card is an indication of preference to next of kin rather than a genuine commitment to donation. Kolber reflects on this challenge in his article on priority allocation from 2003. He maintains that in order “to make a priority incentive system workable, ante mortem decisions to donate must be respected. We cannot grant priority to a registered donor if the effect of that registration can later be trumped by dissenting relatives” [33]. Offering preferential access to a scarce, lifesaving medical resource based on an unenforceable expression of preference is problematic. The individual who has signed a donor card may receive an organ during their lifetime while another equally needy potential recipient is passed over, yet if the preferred recipient is ever able to follow through on their commitment to donate their family may block retrieval. In this sense, priority is being conferred based on empty promises.

There is an asymmetry between the unenforceable promise of organ donation and the guarantee of priority recipient status. This asymmetry is exacerbated by the family veto which renders the commitment to donate meaningless. In this sense, Israel’s policy does not require in-kind reciprocation in return for priority allocation. As Kolber writes: “[A]t no point in time is a human organ ever actually exchanged or promised to be exchanged for something of value. At the time of registration, participants are submitting to the mere possibility of transferring an organ” [33]. Furthermore, the promise of donation can only be fulfilled if authorized by next of kin. Although families rarely override explicit consent to posthumous organ retrieval, the unenforceability of the promise to donate compromises the justifiability of granting priority.

### The policy is discriminatory

Arguments for priority allocation frequently state that organ procurement is a reciprocal enterprise, and those who are willing to receive but not contribute are free riders. As Cronin explains, priority is justified because “a fair concept of justice demands that those who are willing to receive an organ should also be willing to donate one” [34]. Quoting Robert Trivers, who coined the term ‘reciprocal altruism’ in 1971 [35], Israeli heart transplant surgeon Dr. Jacob Lavee claims that the justification for priority allocation is “reciprocal altruism, whereby those in the society who are willing to help others will in turn be helped” and that “the altruist donor benefits because, in time, he ‘is helped in turn’” [36]. Reciprocal altruism describes the exchange of altruistic acts where the net cost to the giver is lower than the net benefit to the recipient. This symbiotic exchange relationship confers long-term mutual benefit to givers and recipients. While the benefit is not immediate, reciprocity will ‘come back around’ over time. Trivers explains that failure to reciprocate (or ‘cheat’ in Trivers’ terminology [35]) is a rational decision where individuals may benefit from altruistic behavior while choosing not to reciprocate. However, the perception of cheating deters others from behaving altruistically, thereby undermining reciprocal altruism altogether. To promote and enforce reciprocity, the advantage of cheating is mitigated by increasing the cost of cheating through ‘moralistic aggression’ - or negative reactions to perceived violations of reciprocity [35]. Landry writes: “Altruism, if supported by ‘strong reciprocity’ that incorporates a propensity to reward altruists and punish the violators of altruistic norms, can operate anonymously in social structures to favour cooperation” [37]. Discouraging free riding and increasing the availability of transplant organs is commendable, however moral blame may not be justifiable or productive. In societies with minority populations for whom the cost of reciprocity is greater than the cost of refusal to behave altruistically, sanctions may be regarded as discriminatory.

In Israel, many Orthodox Jews believe that organ donation is impermissible. While most Israelis accept the importance of transplant sustainability [38] and do not dispute the validity of brain death determination, some regard organs retrieval from brain dead donors as murder - yet permit transplantation. The perceived inconsistency of this position was widely discussed during the implementation of Israel’s priority points system. Lavee and colleagues state: “True believers in the immorality of organ donation after brain death would not be affected by this policy because if organ donation after brain death is wrong, then it should also be wrong for their potential organ donors and hence they should not give or accept an organ” [1]. However, deprioritizing individuals bound by the religious legal judgments of their community is problematic. Kolber explains that limiting access to lifesaving resources is not justified even if the individual holds “inconsistent beliefs” [33], yet accepts that incentives may prompt such individuals to reconsider their position. This prompted reconsideration cannot be expected of those who hold strong views of organ transplantation, including Orthodox Jews who believe that donating one’s organs is impermissible, yet permit using organs that have already been retrieved.

The cost of opting in to the organ pool is greater for Orthodox Jews who do not accept brain death, and this disparity renders priority incentives discriminatory. Furthermore, the perception that the policy is discriminatory lowers public support and participation [39, 40]. Other groups, for example those excluded from donation for medical reasons such as diabetes or HIV, may sign a donor card knowing that they will not be eligible to donate and still receive priority [41]. Heavy smokers, alcoholics, drug abusers, and the morbidly obese can consent to donation and gain priority even if it is unlikely that they will qualify to donate. These conditions do not result in deprioritization even though the diseases associated with them negatively impact organ sustainability. Consent to donation is the only requirement for priority, and the only moral failing deserving of deprioritization is refusal to donate - even the most heinous crimes do not deprioritize or exclude individuals from receiving organs [42]. A response to this seeming inconsistency is that only consent to donation is relevant in determining moral blame and desert: the in-kind reciprocal exchange of organs for organs is a justifiable measure of deservingness of priority. However, in the case of medical exclusion and unhealthy life choices, the promise of donation will remain unfulfilled and is therefore not a reciprocal exchange. If the individual signing the card knows that they will be excluded from donation, this constitutes gaming similar to that observed in Kessler and Roth’s experiment.

The deprioritization of individuals whose religious beliefs explicitly preclude them from donating organs constitutes an infringement on freedom of conscience and may negatively impact trust in the system [43]. As Chandler, et al. explain:

“To the extent that ethnic minorities are less willing to donate, a priority system risks reinforcing some of the potential consequences of minority status that might make a person less likely to donate- feelings of exclusion, discrimination, or distrust […] a policy such as a priority system that emphatically expresses their separateness from the community may just reinforce this feeling” [39].

Those who regard donation after brain death determination as impermissible but are willing to receive transplant organs have been described as free riders, yet this does not justify excluding them from receiving priority. Since the cost of consent is far greater for these individuals, the policy is discriminatory.

A response to this criticism is that those who reject brain death may nonetheless gain maximum priority by donating an organ during their lifetime. However, the cost of signing an unenforceable donor card is lower than the cost of being a live donor. As such, live donation cannot replace consent to posthumous organ donation as a means of accessing priority. In Israel, possible responses to this unequal access may include allocating priority points for other contributions to public health, for example volunteer medic service or repeated blood donation. Daar advances this alternative and draws an analogy to military service, noting that some countries permit conscientious objectors to instead contribute with public service. Daar suggests that those who cannot donate organs could compensate by “playing a role in public education to raise donor awareness, raising funds and so forth” [44]. Singapore’s organ procurement system equalizes access to priority by permitting those who refuse posthumous donation for religious reasons to instead donate their bodies to medical education or research [11]. These alternative contributions could provide means of accessing priority without requiring consent to posthumous organ donation.

The Israeli ADI donor card includes the option to donate only non-essential organs such as kidneys, liver splices, skin, or corneas. The retrieval of these organs would not ‘kill’ the donor and, as previously noted, live kidney donation is a lauded act in the Orthodox community. Stoler and colleagues have noted that media campaigns in Israel have increased donor registration [12]. A campaign directed specifically at disseminating information regarding brain death and donating non-vital organs may encourage greater registration and overcome the gap in access to regular priority. However, it is also possible that such campaigns would not reach their intended audience. Stoler and colleagues noted that although the clerical veto was introduced to accommodate religious communities, this had little impact on registration. The failure of this accommodation to induce registration indicates two potential challenges to the success of information campaigns. The first is that information and accommodations are insufficient to sway ideological non-donors, and the second is that insular communities are not exposed to these campaigns and therefore they are ineffective.

### The information problem

Israel’s priority system faces ethical and practical challenges. Two main challenges to the program’s success are, 1) priority is an insufficient incentive for individuals whose religious beliefs preclude them from donating after brain death determination, and 2) limited access to information and apathy have undermined efforts to educate the public regarding the policy and increase both registration and donation [45].

The first objection was anticipated by numerous thinkers prior to the implementation of Israel’s priority policy. Burkell et al. conducted extensive surveys assessing potential public acceptance of a priority policy in Canada and concluded that “those who are deeply disturbed by the notion of organ donation are unlikely to be motivated by a reciprocity system. The effect of such a system, participants suggest, would be limited to undecided donors without such deep objections, who are therefore susceptible to a self-interest motivation” [40]. Ultra-Orthodox Jews believe that brain death determination does not constitute biological death, and therefore organ retrieval is the proximate cause of death. The expectation that individuals should either renounce their “illusory and irrational” [46] beliefs regarding posthumous bodily integrity and death determination or suffer the penalty of deprioritization is discriminatory. Bramstedt writes, “Since the cost of consent for ideological objectors is much higher than those without strong beliefs regarding donation, priority is not an equally accessible medical benefit” [47]. Steinberg specifically refers to Orthodox Jews as a group for whom priority incentive policies may be discriminatory:

“The definition(s) of death used would have to be precisely stated because some people would not “opt in” if they considered the definition(s) of death used unacceptable. For example, brain death might be an unacceptable criterion for Orthodox Jews […] some adjustment should be made to lessen discrimination against potential organ recipients who were unable to join the “opting in” pool because of established religious views” [48].

The Israeli law incorporates several alternatives to donation of all organs. The first is the clerical veto which stipulates that a clergy member of the family’s choosing must approve organ retrieval. The second is that the family may override the individual’s consent to organ retrieval. A third provision is that the individual may specify that only non-vital organs be retrieved following death determination. The first provision has not increased registration [12], indicating that either objectors were not aware of the provision or that the provision was insufficient to sway ideological objectors. The requirement of consulting a clergyperson also imposes delays that could affect the quality of organs for transplant. The second provision is problematic, as previously discussed, insofar as it permits defeating the express wishes of the decedent and introduces a potentially pernicious loophole. The third provision potentially guarantees equal access to priority while ensuring rapid retrieval of transplant organs. However, public education campaigns have not conveyed the permissibility of this option, as indicated by the continued refusal of some Orthodox Jewish leaders to endorse organ donation and the low rate of consent in these communities [49]. This indicates that media campaigns have limited success in insular communities. While organ donor registration spiked with blitz information campaigns that included placing registration boxes in voting stations during national elections, this effect still may not be sufficient to bridge the gap between need and supply of transplant organs.

Another challenge to the success of information campaigns is low medical literacy, apathy, and public trust. The Haredi population in Israel avoids television, secular magazines and newspapers, Internet, and mainstream radio. Therefore, the “massive multilingual, multimedia educational campaign, designed and aimed at all levels of education in the public […] to gain the most public attention and avoid complaints of discrimination by people who did not participate because they were unaware of the new rules” [1] did not penetrate the community most likely to be negatively impacted by priority allocation. Furthermore, the information campaigns focused on the law itself and not on the religious permissibility of donation. The campaign’s slogan, “Give life, receive life,” may appear coercive to those for whom brain death determination is unacceptable. While the ADI website and organizations such as Arevim provide information about the halachic permissibility of organ donation [50], many Israelis continue to believe that brain death in invalid and that posthumous bodily integrity entails the proscription of organ retrieval.

Despite numerous media campaigns, many Israelis remain unaware of the priority policy or do not understand death declaration, organ procurement, and transplantation [51]. Low medical literacy is not specific to the ultra-Orthodox - misconceptions are also common among populations with lower income and education, and those with low trust in the health care system [52]. This discrepancy contributes to potential discrimination. As Kolber states, “it would be unfortunate for one person to receive less priority than someone else simply because education efforts reached the second person and not the first” [33]. He continues that if priority induced high numbers of donation then even those disadvantaged by ideological refusal or low medical literacy be no worse off than they were previously. However, in Israel there are not enough organs to offset the potential harm of deprioritization. Even though authorization by next of kin reached 62% in 2016, the cadaveric donation rate per million population has remained low and therefore many Israelis remain at risk of deprioritization because they are either ignorant of or do not understand the priority policy.

### Ethical incentives are a form of commodification

The dynamic of organ donation is one of exchange - parts from one body may be traded with those of another. As Kolber points out, the fungible, commodity-like nature of organs is what allows transplantation to be possible whatsoever [33]. The question is whether priority devalues the individual by offering reward in exchange human body parts. Kluge answers that priority “is objectionable from an ethical perspective because it amounts to a de facto institutionalization of payment for organs” [53]. This response relies on the assumption that commodification of body parts is always unethical. However, if commodification of organs is inherently denigrating, then the transfer - or transplantation - of organs could also be regarded as denigrating and unethical. As a 2003 note from the Harvard Law Review elucidates: “In some ways a gift seems to be an even worse instance of value denigration because it does not merely trade one valued thing for another (even if valued in a lower sphere of valuation) but rather trades a valued thing for nothing” [54]. If organs are fungible and valuable because of transplantation, then some forms of commodification will not inherently constitute denigration. Priority incentives, therefore, may be regarded as an ethical form of commodification.

Another possible response to anti-commodificationists is that priority incentives do not commodify organs whatsoever. Rather, since consent to posthumous donation is exchanged for priority points, nothing is being exchanged for organs themselves. As Kolber explains, for commodification to occur, three conditions must be met: “Commodities are typically commensurable (they can be compared and ranked in value), fungible (they can be substituted one for the other), and monetizable (they can be sold and converted into dollars)” [33]. While organs themselves are fungible and may be ‘exchanged’ under certain medical criteria, priority incentives exchange elevated status on the organ recipient waiting list for the promise of donation. These two ‘goods’ are not fungible in the same way that organs are, and priority cannot be sold for money nor can the value of priority be made commensurable through an act of exchange. Furthermore, while organ markets are often regarded as exploitive, Chandler explains that “the congruence of the things exchanged [in priority systems] avoids the offensiveness of describing their value by reference to other tradeable objects” [55]. Thus, while priority has been criticized for commodifying, and therefore denigrating, human bodies, the priority system does not constitute commodification, nor does it perpetuate denigration or exploitation.

The second concern of anti-commodificationists is that incentives erode motivational altruism. Capron charges that an allocation system that prioritizes those who have expressed a willingness to donate “not only commodifies organs in a way that clearly invites a full-fledged market, but it abandons the whole idea of voluntariness that has been at the heart of the transplant system” [42]. Capron and colleagues reiterate this challenge in a whitepaper from 1993 evaluating the potential for introducing priority incentives in the United States. The authors conclude that “perhaps the most important negative aspect of the idea of preferred status is one that it shares with all other forms of inducement: it is likely to be seen by some as inherently compromising the altruism that is a key ingredient of the present voluntary system in which organs are donated as gifts” [56]. However, this perspective assumes that altruism is the sole ethical basis for organ procurement policies. This neglects other sources of justification such as mutual obligation, reciprocity, or a duty of easy rescue, to name a few. Organs may be regarded as a shared communal resource rather than gifts, in which case preserving altruism is unnecessary. Finally, there is no evidence that priority incentives are a slippery slope to full-fledged organ markets. Neither Singapore, which deprioritizes opting out of routine retrieval, nor Israel, which prioritizes opting in to voluntary retrieval, have slid into a full-blown organ market following the implementation of priority incentives. The inducement of priority has not contributed to an increased interest in monetizing organ procurement. Contrarily, the Israeli law has successfully curbed illegal and unethical transplant tourism [Fig. 1]. For these reasons, commodification is not a valid critique of the priority points policy adopted in Israel.

## Is there an alternative?

Israel introduced priority points incentives to promote compliance to cadaveric organ retrieval. However, the policy does not accommodate ideological refusers and has not induced an overall increase in cadaveric donation. In August 2017, an assessment of national donation rates released by the International Registry in Organ Donation and Transplantation (IRODaT) ranked Israel 28 out of 38 reported countries for 2016 [16]. The five countries with the highest rates of donation, Spain, Croatia, Portugal, Belgium, and France, all retrieve cadaveric organs by default. Routine organ retrieval includes all individuals in the donor pool without the requirement of express consent. Opting in procurement policies, such as the one currently in place in Israel, presume refusal to donation and tend to have lower donation rates. Although the Israeli policy has retained presumed refusal to cadaveric organ donation, Siegal’s findings from a 2014 survey demonstrate that close to 70% of Israelis support organ donation [57]. Public acceptance of donation has been further bolstered by widespread media campaigns following the implementation of the Organ Transplant Act. This high rate of acceptance is not reflected in the low rate of cadaveric donation, indicating that although most Israelis would donate their organs after death, very few take positive steps to indicate this preference. In the absence of explicit consent, many next of kin refuse to authorize organ retrieval. The cadaveric donation rate is further negatively impacted by Israel’s stringent brain death determination protocol. Although the priority points policy was intended to overcome the inertia inherent in voluntary organ procurement, and despite a significant increase in next of kin authorization when consent is ascertained, donation rates in Israel remain low. In addition, the priority point policy faces ethical challenges. Routine retrieval with an option to exit the donor pool and permit next of kin to block retrieval, often called ‘soft’ opt out, may resolve many of the ethical challenges while increasing donation. Although a change in default alone is unlikely to increase donation rates [58], implementing routine retrieval in conjunction with other strategies could contribute to transplantation sustainability in Israel. These strategies may include earlier identification of potential donors and improved coordination with families, expanded brain death determination and organ viability protocol, the development of protocol for donation after circulatory death determination, and focused media campaigns conveying the permissibility of cadaveric organ donation in Jewish tradition. Although an in-depth discussion of these potential strategies is beyond the scope of this paper, they have been successful in other countries - particularly in Spain which has been the global leader in organ donation rates for several years [59] - and as such are worth exploring.

## Conclusion

In this paper, I have presented seven challenges to Israel’s current priority policy. Many of them are specific to priority allocation, while others are common to all voluntary procurement policies. Soft opt out, or routine retrieval, respects both individual and family choice by permitting individuals to register refusal while also preserving the family’s ability to block retrieval. As Wilkinson states: “[D]o what the family wants except where it conflicts with the negative right of veto of the individual” [60]. The individual’s right against trespass is upheld by legally binding mechanisms to opt out of the donor pool without penalty, and the family’s wishes not to donate are likewise upheld without permitting the defeat of the decedent’s express wishes. Routine retrieval also resolves the challenge that priority points discriminates against ideological objectors and disadvantages unintentional free riders. The automatic inclusion of all individuals in the organ pool unless they opt out ensures that no one is deprioritized because of their individual beliefs, which constitutes an infringement on freedom of conscience and expression, and likewise protects those who may be disadvantaged by low medical literacy, ignorance of the policy, or simple laziness - none of which should constitute grounds for diminished access to a life-saving medical resource. Although Israel’s Organ Transplant Law was intended to provide an ethical means of narrowing the organ gap, it has not been as effective as hoped and furthermore faces numerous ethical challenges that may not be resolvable. For this reason, it may be time to explore routine retrieval as a possible alternative to the priority points incentive model.

## Abbreviations

DBDD: Donation after brain death determination
PMP: Per million population
